# The duration-energy-size enigma for acoustic emission

**DOI:** 10.1038/s41598-021-84688-7

**Published:** 2021-03-10

**Authors:** Blai Casals, Karin A. Dahmen, Boyuan Gou, Spencer Rooke, Ekhard K. H. Salje

**Affiliations:** 1 Department of Earth Sciences, Cambridge University, Cambridge, UK; 2 Department of Physics, University of Illinois, Urbana, IL 61801 USA; 3 State Key Laboratory for Mechanical Behavior of Materials, Xi’An Jiao Tong University, Xian, 710049 Shaanxi People’s Republic of China

**Keywords:** Materials science, Condensed-matter physics, Ferroelectrics and multiferroics, Phase transitions and critical phenomena

## Abstract

Acoustic emission (AE) measurements of avalanches in different systems, such as domain movements in ferroics or the collapse of voids in porous materials, cannot be compared with model predictions without a detailed analysis of the AE process. In particular, most AE experiments scale the avalanche energy *E*, maximum amplitude Amax and duration *D* as *E* ~ *A*_*max*_^*x*^ and *A*_*max*_ ~ *D*^*χ*^ with *x* = 2 and a poorly defined power law distribution for the duration. In contrast, simple mean field theory (MFT) predicts that *x* = 3 and *χ* = 2. The disagreement is due to details of the AE measurements: the initial acoustic strain signal of an avalanche is modified by the propagation of the acoustic wave, which is then measured by the detector. We demonstrate, by simple model simulations, that typical avalanches follow the observed AE results with *x* = 2 and ‘half-moon’ shapes for the cross-correlation. Furthermore, the size S of an avalanche does not always scale as the square of the maximum AE avalanche amplitude *A*_*max*_ as predicted by MFT but scales linearly *S* ~ *A*_*max*_. We propose that the AE rise time reflects the atomistic avalanche time profile better than the duration of the AE signal.

## Introduction

Avalanches commonly occur during deformation and failure of materials^1–25^. They dominate the hysteretic behaviour of domain switching in electric, magnetic or strain fields. They also occur in scenarios where local restructuring occurs, such as the collapse of voids in porous materials. Here we focus on ferroic switching as the defining feature that contributes to the classic Barkhausen noise^26–31^. The energy of avalanches in Barkhausen noise is partially released by elastic waves during acoustic emission, AE. As AE measurements have unsurpassed sensitivity they became the method of choice for the investigation of field induced changes in ferroics and are hence at the heart of current research into the dynamics of switching processes^2,13,14,17–22,30,32–36^. Here we show why some results of AE deviate from the predictions of mean field theory, MFT^14,37^.

The following parameters are typically measured during AE experiments: the energy *E* of an avalanche, the time evolution of the AE amplitudes *A*_*AE*_(*t*)*,* the maximum AE amplitudes *A*_*max*_ per avalanche, their duration *D* and various correlations between avalanches like waiting time, aftershocks^38^ etc.… Other parameters are obtained indirectly, like the size *S* by integrating *|A*_*AE*_(*t*)*|* over time *t* during the progression of the avalanche. The fundamental assumption is then that these quantities can be equated with the fundamental avalanche parameters (i.e. of the behaviour of the avalanche source). These parameters are *E, V*(*t*)*, V*_*max*_*,* and duration *T*. Similarly, the size *S* is derived from model simulations by integration over *V*(*t*)*,* this function can then be compared with the AE size function. We use the common nomenclature of the AE literature and add a subscript *AE* whenever there is a danger that the reader confuses the quantities of the AE experiment and the equivalent (atomistic) avalanche parameter. The non-equivalence between *E*_*AE*_ and *E, A*_*AE*_(*t*) and *V*(*t*)*, A*_*max*_ and *V*_*max*_, and the AE duration *D* and its equivalent duration *T* of the atomic avalanche is at the heart of this study.

**Figure 1:**
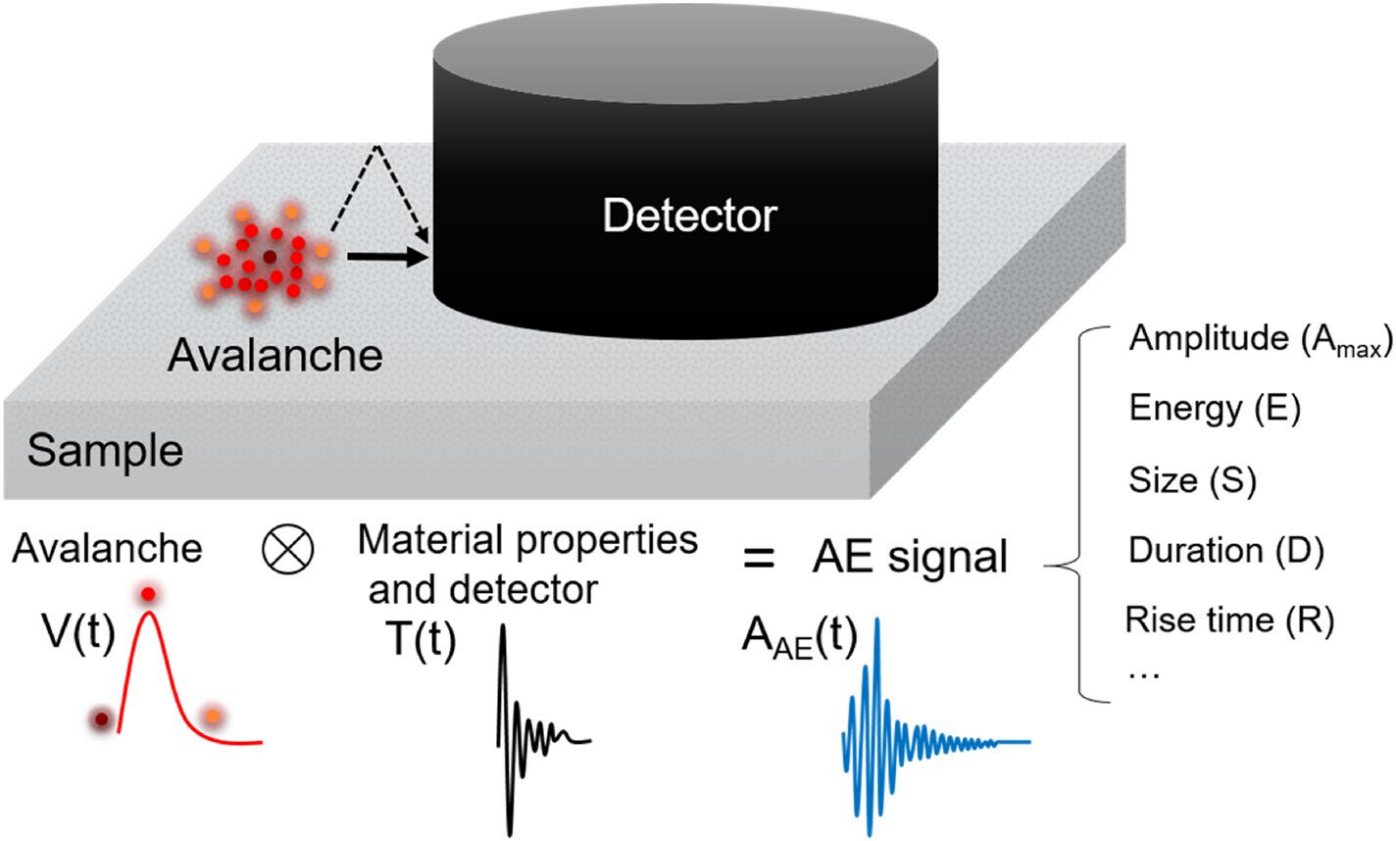
Schematic representation of the composition of an avalanche signal in acoustic emission experiments. The size of the sample and the detector is some 5 mm, the detector covers often a large part of the sample. During local switching, an avalanche with *V*(*t*) emits a strain signal (i.e. the source function) which propagates through the sample and is eventually measured by the detector. During the propagation, the signal generates the ringing of the sample and is modified by elastic wave reflections on surfaces, scattering on lattice imperfections. The profile of a source delta function would generate *T*(*t*)*,* the so-called transfer function. The measured AE profile *A*_*AE*_(*t*) is described in this paper as the convolution of the source function with the transfer function.

The amplitude *A*_*AE*_(*t*) is an experimental quantity directly measured in an AE experiment, namely the amplitude of the strain wave arriving at the AE detector (Fig. 1). The local process of the avalanche nucleation and progression produces local strain amplitudes which is denoted *A*(*t*). *A*(*t*) is the local strain amplitude at the time *t* and represents the change of the atomic configuration at time t. It hence represents a ‘rate of change’ which we call in this paper *V*(*t*). The atomic interpretation of *V*(*t*) as ‘velocity’ of the advancing front of an avalanche is appropriate if the avalanche is a fairly compact region while nucleation and fractal regions do not have such a simple geometrical interpretation. In these cases, *V*(*t*) is understood as a ‘displacement rate’ when the objects are spatially distributed or fractal. In accordance with convention, we still use the symbol *V*(*t*) bearing in mind this more general ‘rate’ definition. In particular, the relevant patterns in ferroics are extremely complex and only occasionally compact. We use the symbol *V*(*t*) with the understanding that *V*(*t*) does not just mean a simple front propagation as, e.g., described in the Avrami–Ishibashi approach^39,40^.

The analysis of AE spectra is typically based on their comparison with model simulations^4–7,12,14,18,19,23^. Most model parameters were initially borrowed from research into Earth Quake statistics [e.g.^41^] so that the nomenclature was historically taken from this field. The fingerprint for critical avalanches is that the probability distribution functions (PDF) of *E*_*AE*_*, A*_*max*_*,* and *D* are power law distributed with exponents *ε,* τ′*,* and *α*, respectively. Here the energy is derived from the AE spectrum of *A*_*AE*_(*t*) by $E_{AE} = \mathop \smallint \limits_{0}^{{D_{AE} }} A_{AE} \left( t \right)^{2} dt$. Correlations exist between these parameters which also follow power laws with $E_{AE}$ ~ *A*_*max*_^*x*^ and *A*_*max*_ ~ *D*_*AE*_^χ^^30^.

Experimentally, the power law exponents *ε* and τ′ are typically measured over many decades and our knowledge of any systematics of the dynamic properties of energies and amplitudes is rather good. The same is not true for the duration *D*. There are very few AE measurements available to determine *α* with reasonable accuracy^17,42,43^. The PDF of the duration *D* often follows a power law in some approximation for long durations, while it shows either constant or exponential distributions for short durations. ‘Half-moon’ shapes are found when plotting the durations as function of the maximum amplitudes, *D*(*A*_*max*_)*,* and duration as function of the avalanche energy, *D*(*E*)^17,30,44^*.* As the integration to determine *E*_*AE*_ covers the duration of the AE signal, the correlation between the energy *E*_*AE*_ and the maximum amplitude of an avalanche *A*_*max*_ as described by the correlation exponent *x*, is equally enigmatic.

The enigma, which we address in this paper, is the scaling between the AE energy and the AE maximum amplitude $E_{AE}$ ~ *A*_*max*_^*x*^. This relation is often the starting point for further analysis of the avalanche mechanism. Experimentally, *x* = 2 is found in systems that contain distributions of domain movements that are otherwise consistent with MFT. AE measurements for BaTiO_3_^30^ are a key example for *x* = 2 while *x* = 3 would be consistent with MFT simulations. To the best of our knowledge, only the exponent *x* = 2 has been reported in AE experiments. Exponents *x* = 3 or intermediate values between 2 and 3 were discussed, but not observed experimentally, by Vives et al.^41^. These authors clearly identified the discrepancy between *x* = 2 and *x* = 3 but offered no reason. We rationalize this observation and argue that avalanche exponents are modified by details of the AE experiments^45^.

The characteristic parameters for the energy (*E*), maximum amplitude (*A*_*max*_), size (*S*), duration (*D*) and rise time (*R*) are evaluated by the following definitions from the time evolution of the $\Psi \left( t \right)$ signals (the so-called ‘jerk spectrum’, this terms is used both for AE spikes and also for atomic avalanche anomalies) which hence becomes *V*(*t*) before the convolution and *A*_*AE*_(*t*) after the convolution :

1 $$A_{max} = max\left( {\left| {\Psi \left( t \right)} \right|} \right)$$

2 $$D = t(\left| {\Psi \left( {t \to t_{n} } \right)} \right| > \Psi_{th} )$$

where $\Psi_{th}$ is a threshold of $\Psi \left( t \right)$ and *t*_*n*_ is the waiting time.

3 $$R = t\left( {\left| {\Psi \left( t \right)} \right| = {\text{Maximum}}} \right)$$

4 $$E = \mathop \smallint \limits_{0}^{D} \Psi \left( t \right)^{2} dt$$

5 $$S = \mathop \smallint \limits_{0}^{D} \left| {\Psi \left( t \right)} \right|dt.$$

The PDFs of these magnitudes are power laws (e.g. for energy $PDF\left( E \right)\sim E^{ - \varepsilon }$). Here we use the exponent $\varepsilon$ for $E$, $\tau ^{\prime}$ for $A_{max}$ , $\tau$ for $S$, $\alpha$ for $D$ and $\rho$ for $R$. The correlations between these magnitudes are defined as $E \sim A_{max}^{x}$, $S \sim A_{max}^{\gamma }$, $A_{max} \sim D^{\chi }$ and $A_{max} \sim R^{\xi }$.

## Sample ringing and average avalanche profile

AE spectroscopy is characterised by a close proximity of the sample and the detector. Both have very similar dimensions and are closely coupled. It appears impossible to analyse an emitted wave profile, as sometimes done in geophysics and other acoustic investigations of large bodies, by either inverse or direct methods^46^. Instead, we consider in this paper a simple approach to estimate the influence of wave propagation on the scaling of the typical AE parameters. We cannot investigate the actual waveform of the triggering event nor do we analyse the sample-shape dependence of the AE signal, still we derive a simple interpretation of the AE scaling *x* = 2.

We first consider acoustic emission of a ferroic material that originates from the propagation of domain boundaries and domains during switching^30^. When domain walls hit the sample surface or when domain walls intersect or annihilate, they emit a primary strain signal that incites the traveling acoustic wave. Similarly, crack propagation^47,48^ and porous collapse^17,19^ generate equivalent AE signals. The signals are then measured by acoustic detectors and analysed electronically. The key characteristic of AE is that it does not measure the acoustic collision directly but measures the macroscopic vibration (the so-called ringing) of the sample. Such ringing phenomena in the 10^4^–10^7^ Hz region were thoroughly investigated in the field of acoustic resonance spectroscopy^49–55^. The frequency of the ringing is determined by the elasticity of the sample and is only weakly influenced by the acoustic wave emitted by the domain switching process.

In order to demonstrate the modifications of the initial source function we first consider a simple toy model. We use the theoretical averaged source function *V*(*t*) which would be measured if the time resolution of the experiment were extremely poor. In this case, many local events overlap and initiate the ringing of the sample. This approach has the advantage to clarify the various parameters analytically; a full simulation is presented in the next paragraph. We use the averaged source function^37,56^ with the time variable *t*

6 $$\it {\text{V}}\left( {\text{t}} \right) = ate^{{ - bt^{2} }}$$

The maximum amplitude A_max_ of the source signal V(t) scales with parameters $a$ and $b$ as,

7 $$A_{max} \sim ab^{{ - \frac{1}{2}}}$$

and the rise time called when the maximum amplitude is reached

8 $$t|_{{A_{max} }} \sim b^{{ - \frac{1}{2}}}$$

The size of the avalanche is defined as

9 $${\text{S}} = \mathop \smallint \limits_{0}^{ + \infty } \left| {V\left( t \right)} \right|dt\sim {\text{a}}/{\text{b}}$$

The traveling wave is then approximated by the convolution of the source signal with the transfer function. This convolution is expressed by the integral

10 $$\it {\text{A}}_{{{\text{AE}}}} ({\text{t}}^{\prime } ) = \mathop \smallint \limits_{0}^{ + \infty } V\left( t \right)T\left( {t^{\prime} - t} \right)dt$$

This integral extends over the full time, which in an experimental situation means the full duration of the source function. We tested several transfer functions (see supplemental material section 1). In our toy model we use a exponentially damped sinusoidal which is compatible with typical experimental AE profiles^42,45,57,58^:

11 $$T\left( t \right) = \cos \left( {wt} \right)e^{ - qt}$$

where *w* is the frequency and *q* is a damping parameter. Figure 3a shows the source function and its convolution with the transfer function. We also explored a Gaussian decay (supplementary information section 1 and 3) which is often used to describe resonance ultrasound spectroscopy (RUS) signals^59^, the convolution shows very similar behaviour to the simple exponential decay.

**Figure 2:**
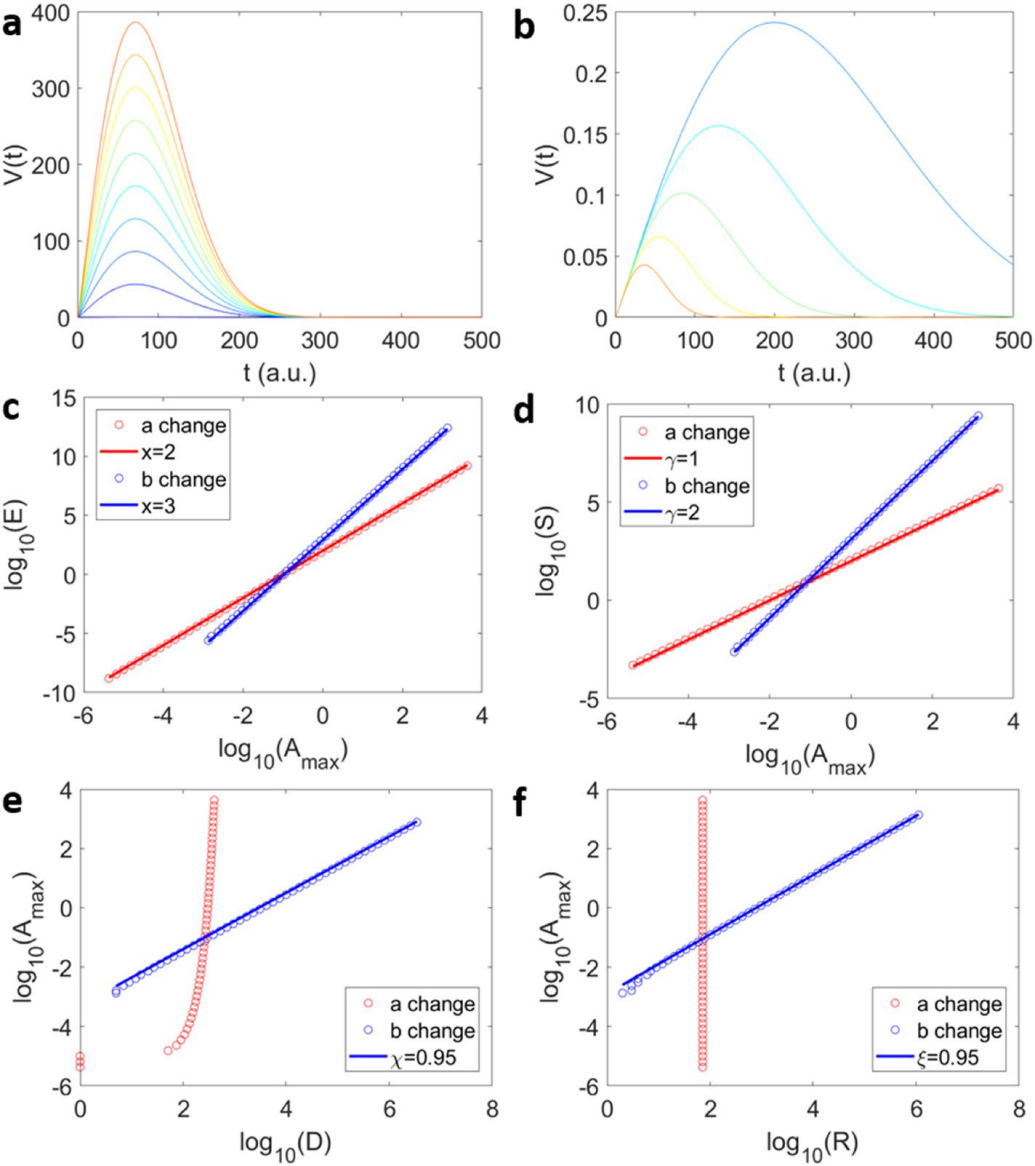
Scaling relations for the averaged source function. Shapes of source functions ${\text{V}}\left( {\text{t}} \right) = ate^{{ - bt^{2} }}$ with different parameters *a* (**a**) and *b* (**b**), as shown, *a* changes the amplitude and *b* modifies both duration and amplitude. Panels (**c**–**f**) show scaling relations before convolution for $E \sim A_{max}^{x}$ (**c**), $S \sim A_{max}^{\gamma }$(**d**), $A_{max} \sim D^{\chi }$(**e**) and $A_{max} \sim R^{\xi }$ (**f**) when the model parameters a (red circles) and b (blue circles) are varied.

The source function is parameterised by the non-universal constants $a$ and $b$ denoting the amplitude and the position of the maximum (i.e. the width of the distribution), respectively. A typical example for the variation of the source function depending on its width is shown in Fig. 2a, b. The energy *E* is calculated as the integral over the squared temporal amplitude.

12 $$\begin{aligned} E & = \mathop \smallint \limits_{0}^{ + \infty } V\left( t \right)^{2} dt = \mathop \smallint \limits_{0}^{ + \infty } a^{2} t^{2} e^{{ - 2bt^{2} }} dt \\ & = \frac{{\sqrt 2 a^{2} }}{{8b^{\frac{3}{2}} }}\mathop \smallint \limits_{0}^{ + \infty } u^{{\left( {\frac{3}{2} - 1} \right)}} e^{ - u} du{ }\sim a^{2} b^{{ - \frac{3}{2}}} \\ \end{aligned}$$

**Figure 3:**
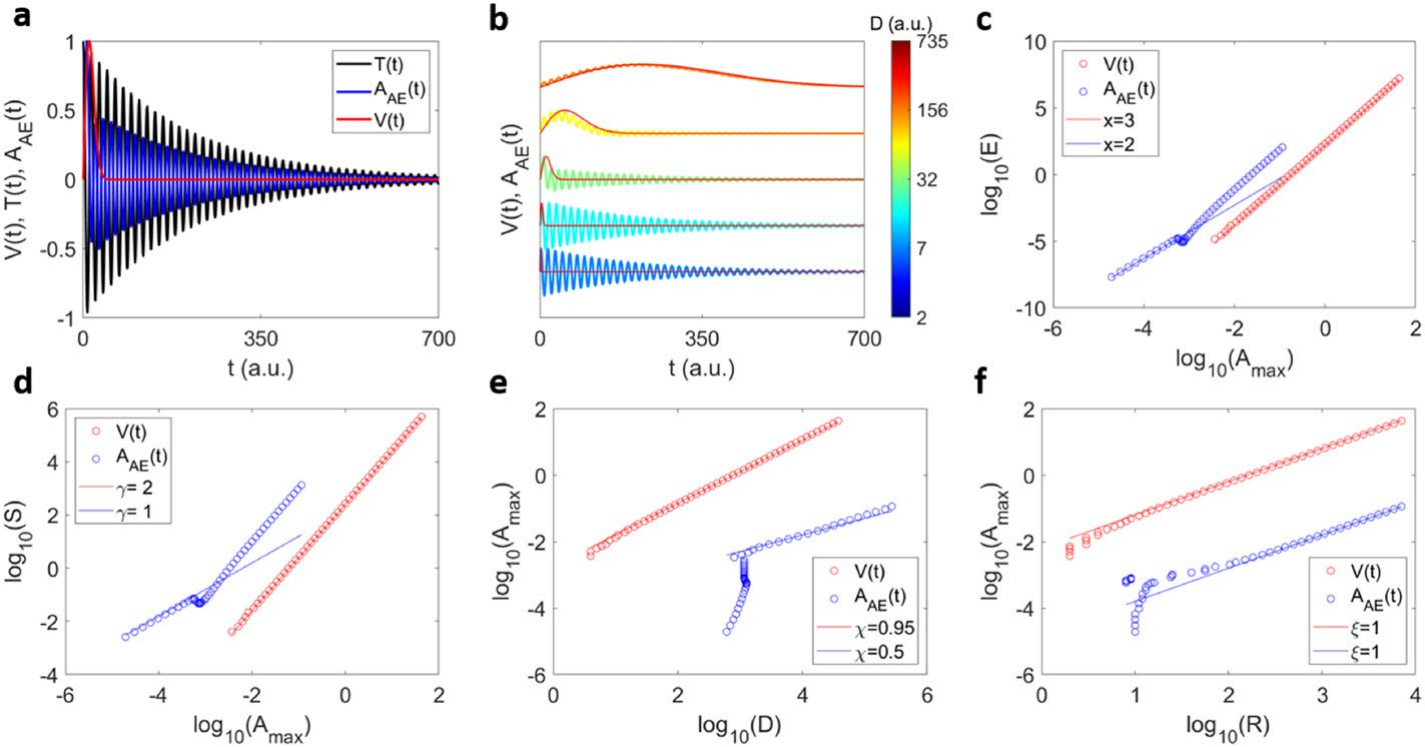
Scaling relations for the averaged source function before and after convolution. (**a**) Example of the convolution (in blue) of an avalanche profile *V*(*t*) (in red) with a transfer function ($\cos \left( {wt} \right)e^{ - qt}$ with *w*=0.5 and *q* = 5 × 10^−4^ with time unit normalized to 1 (in black). (**b**) Examples of convoluted avalanche profiles for different durations (blue to red when decreasing the **b** parameter) and the original profiles (in black). Both convoluted and elementary profiles were normalized with respect to their maximum amplitudes. The scaling correlations $E \sim A_{max}^{x}$ (**c**), $S \sim A_{max}^{\gamma }$ (**d**), $A_{max} \sim D^{\chi }$(**e**) and $A_{max} \sim R^{\xi }$ (**f**) for the original *V*(*t*) profiles (red points) and after the convolution for *A*_*AE*_(*t*) (blue points).

Remembering (*a*/*b*^0*.*5^)^2^ * b^−0.5^ = *A*_*max*_^2^ * t ($A_{max}$) shows that *E* is proportional to *A*_*max*_^2^ multiplied by the time when the maximum amplitude occurs. A constant model parameter *a* leads to *E* ~ *A*_*max*_^3^ as shown in Fig. 2c. The size can be deduced similarly and shows *S* ~ *A*_*max*_^2^ in Fig. 3b. However, if we keep the *b* parameter constant and change the *a* parameter then the scaling becomes *E* ~ *A*_*max*_^2^ (Fig. 2c) and *S* ~ *A*_*max*_ (Fig. 2d). This difference stems from the duration of the source function. The duration is defined by the time to reach a given threshold of the amplitude, whereby this threshold is constant for all *A*(*t*)*.* When changing the amplitude (*a* parameter) the duration reaches an asymptotic limit (‘half-moon’ shape in Fig. 2e) and since the position of the *A*_*max*_ is unchanged, the rise time is constant (Fig. 2f). In the opposite scenario, when changing the *b* parameter, the duration and the rise time scales linearly with *A*_*max*_. We can use *A*_*max*_ to compare the different scaling between parameters because *A*_*max*_ does not depend on the duration of the *V*(*t*) profile.

We now consider the convolution in Eq. (10) between the source function *V*(*t*) and the transfer function *T.* With an ansatz $T = \cos \left( {wt} \right)e^{{ - qt^{2} }}$ we calculate *A*_*max*_*, E, S, D*, and the rise time *R* of the convoluted *A*_*AE*_(*t*) waveform using the Eqs. (1–5). Figure 3a shows an example of the convolution between the source function and the transfer function. The waveform of the convoluted function *A*_*AE*_(*t*) changes by changing the *b* parameter as shown in Fig. 3b. For short durations, the convolution shape is closer to the shape of the transfer function and for large durations the shape is more similar to the source function. The scaling of *E*_*AE*_ with *A*_*max*_ shows equally two tendencies (Fig. 3c): for small values of *A*_*max*_ the scaling is *E* ~ *A*_*max*_^2^ and for high values it becomes *E* ~ *A*_*max*_^3^. Similarly, the scaling of *S*_*AE*_ with *A*_*max*_ shows *S* ~ *A*_*max*_ for low amplitudes and *S* ~ *A*_*max*_^2^ for large amplitudes (Fig. 3d). This change of scaling is caused by the large variability of the duration *D* in the convoluted *A*_*AE*_(*t*) waveforms (Fig. 3e). For short source function durations, the convoluted duration is dominated by the duration of the transfer function, thus, converging to an asymptotic value. In this regime, the source function effectively determines the maximum amplitude *A*_*max*_ of the convolution but does not change the duration *D*. For large source function durations, the convoluted duration increases with the maximum amplitude of the source function and changes the integrals to calculate *E*_*AE*_ and *S*_*AE*_, which modify their scalings with respect to *A*_*max*_. This behaviour differs from the scaling of the rise time *R* which does not change with variable *A*_*max*_ after convolution (Fig. 3f). In this toy model we hence observe both scenarios, *x* = 2 and *x* = 3, depending on the duration of the local strain signal: if the source signal is short (e.g. delta function) the AE signal is identical or close to the transfer function and we find *x* = 2. For long local events we find *x* = 3.

## Model simulations with transfer functions

### The collapse model

We now consider more realistic source functions and ask whether source functions alone, without the interference of the transfer function, can generate the observed *E*_*AE*_ ~ *A*_*max*_^2^ scaling. For this purpose, we construct a simple atomic model based on the mechanism leading to porous collapse. In this model we choose random sites for the collapse and a feedback that stipulates that the next step depends on the collapsed regions multiplied by a probability chosen from a probability distribution function *f*(*t*) as

13 $$V_{a} \left( t \right) = f\left( t \right)V_{s} \left( {t - 1} \right).$$

**Figure 4:**
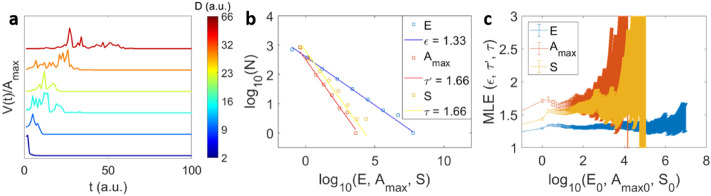
Avalanches of the collapse model. (**a**) Examples of normalized avalanche profiles *V*(*t*)*/A*_*max*_ with different durations (blue to red means increasing the duration) for simulations of the model of Eq. (13). PDF (**b**) and maximum likelihood exponent (MLE) (**c**) of the energy (*E*), maximum amplitude *A*_*max*_ and size *S* for the model of Eq. (13).

Here *V*_*s*_(*t* − 1) is the is number of collapsed sites at *t* − 1 and *f*(*t*) is a Gaussian function centered at zero with half width **w**. The product defines the number of attempted collapsing sites *V*_*a*_(*t*) that will be placed randomly in space. The sites will successfully collapse if they do not overlap with previously collapsed sites. Thus, the avalanche profile *V*(*t*) is defined by the collapsed sites per time *V*_*s*_(*t*). The initial condition is the collapse of a single site ($V_{s} \left( {t = 0} \right) = 1$) that is randomly chosen. When an avalanche ends because *V*_*s*_(*t* − 1) = 0, the next avalanche is chosen to start at a new random site with *V*_*s*_(0) = 1 in an empty frame. The half width of *f*(*t*) is chosen as **w** = 1*.*4 in our simulations, more general conditions will be discussed in a forthcoming paper. This model is similar to the mean field approximation of the non-equilibrium random field Ising model with power law distributed spin flip avalanches at a critical width of the distribution of random fields^60–63^. Finite-size effects have an impact on the cut-off of *E, A*_*max*_*, S* etc. but not on the correlation between these quantities. This is caused by a direct impact of the overlap between attempted sites *V*_*a*_(*t*) and previously collapsed sites *V*_*s*_(*t* − 1). The widest scaling interval is found when the overlap is reduced to zero. We hence use the Eq. (13) without placing actual sites in space and accepting all sites to collapse successfully *V*_*a*_(*t*) = *V*_*s*_(*t*) = *V*(*t*). Here we use avalanches profiles produced by the equation *V*(*t*) = *f*(*t*)*V*(*t* − 1). Examples of the computed avalanche profiles are shown in Fig. 4 for different durations. The average avalanche profile^11,37^, generated by this model, is similar to the initially used average avalanche profile (supplementary information section 5).

The PDFs are shown together with the maximum likelihood profiles of *E*, *A*_*max*_ and *S* in Fig. 4a,b. The PDFs exhibit a good power law behavior with the exponents *ε* = 1.33 (for *E*), α = 1.66 (for *A*_*max*_) and τ = 1.66 (for *S*).

**Figure 5:**
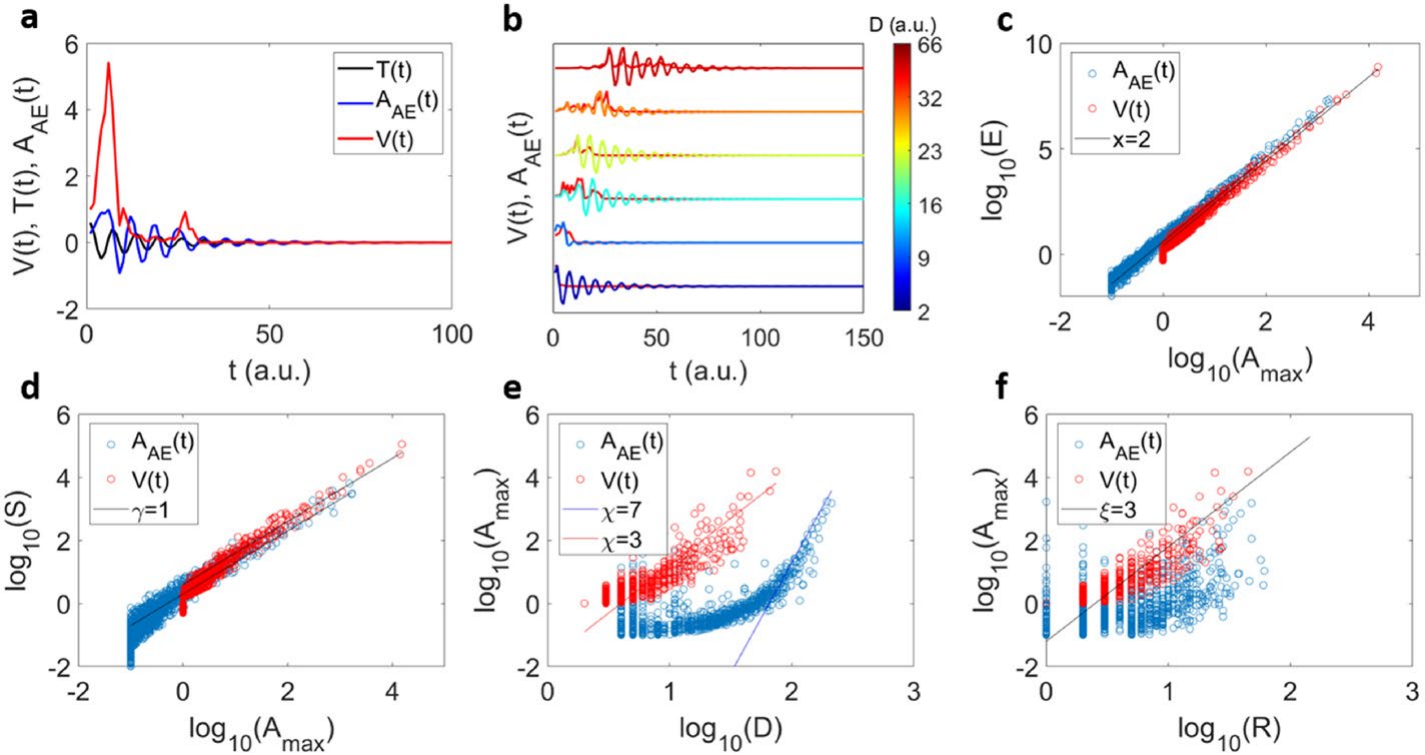
Scaling relations of the collapse model before and after convolution. (**a**) Example of convolution leading to *A*_*AE*_(*t*) with an avalanche profile *V*(*t*) (in red) and a transfer function *T*(*t*)* = *$\cos \left( {wt} \right)e^{ - qt}$ (in black). (**b**) Examples of 6 convoluted avalanche profiles for different source functions *V*(*t*) with different durations, and the same transfer function. Both convoluted and original profiles are normalized with respect to their maxima. (**c**–**f**) scaling relations $E \sim A_{max}^{x}$ (**c**), $S \sim A_{max}^{\gamma }$ (**d**), $A_{max} \sim D^{\chi }$(**e**) and $A_{max} \sim R^{\xi }$ (**f**) for the original *V*(*t*) profiles (red points) and after the convolution *A*_*AE*_(*t*) (blue points).

The correlations between the avalanche magnitudes are given in Fig. 5 (red circles in c–f). The scaling between *E* and *A*_*max*_ shows $E\sim A_{max}^{2}$ (Fig. 5c). The scaling between *S* and *A*_*max*_ follows the relation $S\sim A_{max}$ (Fig. 5d). The duration of *V*(*t*)*,* computed as the time when *V*(*t*) crosses a threshold, scales with *A*_*max*_ as shown in Fig. 5e. The rise time scales in the same way as the duration (Fig. 5e,f). Our simple model exhibits the half-moon profile of the correlation between the duration and the maximum amplitude.

We now explore the scaling after convolution to *A*_*AE*_(*t*) with different transfer functions. As an example, we consider again the transfer function $T = \cos \left( {wt} \right)e^{ - qt}$, where *w* is the frequency and *q* a damping factor with *w* = 1(1*/t*) and *q* = 10^*–*7^ (1*/t*^2^). Other transfer functions are shown in the Supplementary Material section 1, 3 and 4. The Fig. 5a shows an example of a single avalanche profile, the transfer function and the result of the convolution. Different AE signals *A*_*AE*_(*t*) are shown in Fig. 5b for the same transfer function but different durations. The scaling between *E, A*_*max*_ and *S* remains unperturbed by the convolution as shown in Fig. 5c,d. However, the scaling between *A*_*max*_ and duration is different after convolution and strongly depends on the chosen transfer function (see supplementary information section 2, 3 and 4) and the threshold that is used to define the duration of *A*_*AE*_(*t*). The time evolution of *A*_*AE*_(*t*) is dominated by the decay of the transfer function. We now calculate the rise time, defined as the time to reach *A*_*max*_*.* The relation between the rise time *R* and the duration *D* is proportional *R* ~ *D* for the *V*(*t*) profiles and it is preserved after the convolution, as shown in Fig. 5f and directly for the convoluted profiles in Fig. 5b. The model is similar to spin-based models, such as the non-equilibrium random field Ising model^37,60–63^.

### The slip model

**Figure 6:**
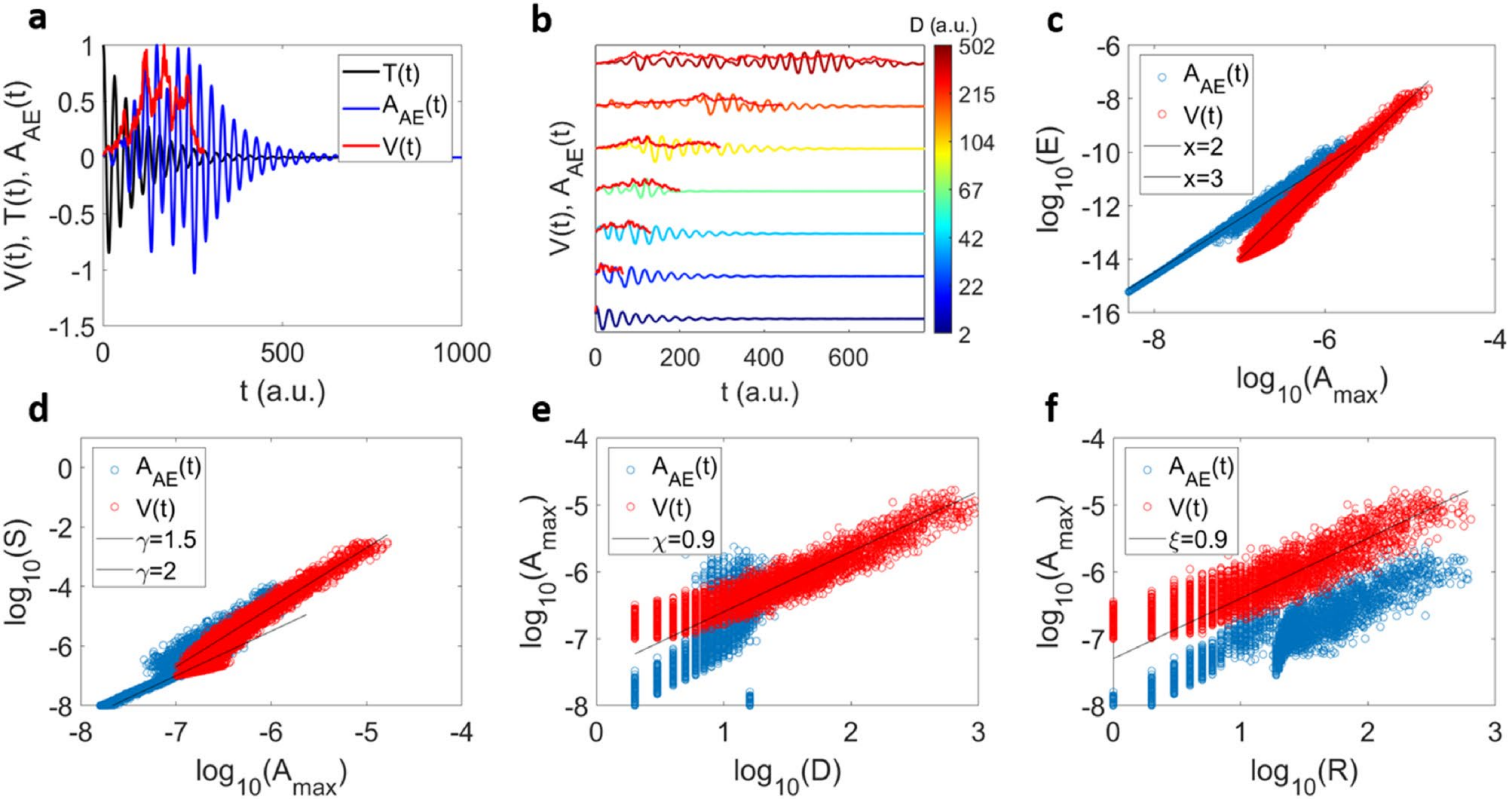
Scaling relations of the slip model before and after convolution. (**a**) Example of a convolution leading to *A*_*AE*_(*t*) (in blue) from an avalanche profile *V*(*t*) (in red) and a transfer function *T*(*t*)* = *$\cos \left( {wt} \right)e^{ - qt}$ (in black). (**b**) Examples of 6 convoluted avalanche profiles for different source functions *V*(*t*) with different durations, and the same transfer function. Both convoluted and original profiles are normalized with respect to their maxima. (**c**–**f**) scaling relations $E \sim A_{max}^{x}$ (**c**), $S \sim A_{max}^{\gamma }$ (**d**), $A_{max} \sim D^{\chi }$(**e**) and $A_{max} \sim R^{\xi }$ (**f**) for the original *V*(*t*) profiles (red points) and after the convolution *A*_*AE*_(*t*) (blue points). For e) and f) the exact exponent values in mean field theory are *Χ*=1 and *ξ*=1.

We compare these results with mean field simulations of a simple slip model^23^. In contrast to the collapse model, the slip model takes into account the long-range character of the elastic interactions between weak spots in a slowly sheared solid. While the collapse model is more empirical in spirit, the mean field model is a coarse-grained model that is based on the physical dynamics on scales as small as individual weak spots in the material. It is again a threshold model, and the long-range interactions between the slips in the material enable one to compute analytical solutions for the universal scaling behavior of the slip statistics. Renormalization group methods have been used to show that this scaling behavior is the same as slips in slowly deformed solids with slip localization, such as nanocrystals, bulk metallic glasses, rocks, jammed granular materials, friction and earthquakes^23,25,60–63^. The model assumes a solid that consists of *N* interacting cells, each of which can slip by a random amount when the local stress reaches a local stress threshold. When a cell slips the released stress is equally redistributed to the other cells, which can trigger other cells to slip also in a slip avalanche. A slip avalanche ends when the stress at all cells is below the local failure stress. Then the external loading stress is slowly increased until the next cell slips, triggering the next avalanche. In its simplest version the model predicts power law distributed avalanche sizes, and durations. The time profiles of the avalanche propagation speeds (averaged over all avalanches of the same size or duration) follow simple scaling functions that can be computed analytically^23^. Many predictions for the scaling properties of the model are given in^23,25,60–63^. There are related spin flip models, such as the non-equilibrium random field Ising model, which give similar scaling results^60–62^, and they are likely even more closely related to the model introduced here because of their notable disorder dependence. Here, the source time functions are extracted from the slip model and convolved with the transfer function *T*(*t*). The results are shown in the Fig. 6.

Following the same steps as previously, we plot the transfer function *T*(*t*) with an example of a source function *V*(*t*) and the correspondent convolution *A*_*AE*_(*t*) in Fig. 6a. Figure 6b shows some examples of *V*(*t*) and the corresponding *A*_*AE*_(*t*) for increasing durations. The correlation $E \sim A_{max}^{x}$ exhibits *x* = 3 for the local strain signal *V*(*t*) and *x* = 2 for the measured AE signal *A*_*AE*_(*t*) (Fig. 6c). Similarly, the scaling relation $S \sim A_{max}^{\gamma }$ shows *γ* = 2 for *V*(*t*) and *γ* = 1*.*5 for *A*_*AE*_(*t*). This change of the scaling relation exponent is expected as shown previously for the average source function ${\text{V}}\left( {\text{t}} \right) = ate^{{ - bt^{2} }}$ (see Fig. 3). The duration scaling with *A*_*max*_ before the convolution is clearly different for the collapse model and the slip model, after convolution the initial scaling is almost lost. Only the rise time *R* preserves qualitatively the scaling after convolution (Fig. 6f).

## Conclusion

We have shown that the measured AE spectrum, i.e. the macroscopic jerk spectrum of a sample, does not reflect the initial avalanche distribution *V*(*t*) nor the predictions of mean field theory. There are two main reasons. Firstly, the measured AE is modified by the transfer function in a well-defined fashion. This modification is most notorious if the time and frequency scales of the initial avalanche formation (some microseconds in many cases) differs greatly from the inverse frequency of the sample ringing and hence the transfer function. Ringing times are typically between several microseconds and some milliseconds. This time scale depends on the sample size (decreasing transfer time with decreasing sample size) and the nature of the initial avalanche. Crack propagation and dislocation dynamics are fairly similar in their local duration *D* while ferroelectric and ferroelastic domain movements are often much slower. Nevertheless, the general rule is that the transfer function shifts exponents to *x* = 2 while *x* = 3 has never been observed experimentally.

Comparing the toy model with the more realistic profiles of the transfer function highlights the reasons. The average profile of the toy model is very broad. In contrast, the simulated avalanche profiles and experimental profiles show few very sharp peaks, often only one dominant peak. The convolution with the transfer function then yields the transfer function as *A*_*AE*_. All information about the initial duration of the source event and its line profiles is lost and only the transfer function is measured. This seems to be the fate of most AE spectra so that a detailed analysis of the observed jerk profiles only reveals information about the transfer function and says little about the local avalanche mechanism. The main information is hence the parameter *x*: *x* = 2 means that the local process is fast compared with the sample ringing, *x* > 2 means that some information about the local time scale could be obtained from AE spectra.

The observation *x* = 2 does not mean that the avalanche process does not follow the predicted mean field scaling. The observed duration is usually not the correct time scale because it is strongly modified by the transfer function. If the spectrum *V*(*t*) contains narrow peaks, then the integral is determined by the integral over the peak area and not by the much wider integration interval over the duration of the avalanche. This effect is very common in ferroelastics where a domain collapses or scatters with another domain. After one massive event, there are long-term relaxations, which contribute to the duration *D* but contain very little energy. In this case we find that *x* = 2.

The third fundamentally important result is that the duration is in all cases a very complex quantity, which cannot be approximated easily. In contrast, we find that the rise time R represents the avalanche formation and propagation much better (supplementary information section 7). It is close to the local rise time and is less polluted by the effect of the transfer function.

Different models for avalanches statistics yield similar macroscopic behaviour while some characteristic differences occur^23,24^. The power law distributions for energy and sizes are invariant (supplementary information section 5), however. The main differences stem from the role played by the duration. The ‘half-moon’ shape of *A*_max_*(D)* and *A*_max_*(R)* is well reproduced in the collapse model but much less in the slip model. This result points to the usefulness of the duration and specially rise time as AE avalanche characteristics, which allow at least a partial distinction between different atomic avalanche mechanisms. Further work on the distinction between avalanches models and their consequences for AE measurements are planned.

## Supplementary Information

**Supplementary Information**.

## Data Availability

The data that support the findings of this study are available from the corresponding author upon reasonable request.
