# Supplementary material for: The duration-energy-size enigma for acoustic emission
Source: Sci Rep. 2021 Mar 10;11:5590. doi: 10.1038/s41598-021-84688-7 (PMC7947008; doi:10.1038/s41598-021-84688-7)
Supplement: Supplementary file 1 — Supplementary Information. [file 41598_2021_84688_MOESM1_ESM.pdf]

SUPPLEMENTARY MATERIAL

The Duration-Energy-Size Enigma for Acoustic Emission

Blai Casals[1,*], Karin A. Dahmen[2] , Boyuan Gou[3], Spencer Rooke[2], Ekhard K.H. Salje[1]

[1] Department of Earth Sciences, Cambridge University, Cambridge UK
[2] Univ Illinois, Dept Phys, Urbana, IL 61801 USA
[3] Xi An Jiao Tong Univ, State Key Lab Mech. Behav. Mat., Xian 710049, Shaanxi, Peoples R China

* Correspondence to blaicasals@gmail.com

1. Convoluted avalanche profiles from different transfer functions

Fig. S1 shows the convolution from a randomly selected avalanche profile *V(t)* of the collapse model with three different transfer functions. We consider $T(t) = \cos(wt)\, e^{-qt}$ as the response of a damped harmonic oscillator, $T(t) = \cos(wt)\, e^{-qt^2}$ where the damping is a Gaussian function instead of an exponential function. The Gaussian function accounts for wave reflections (with a Gaussian distribution) on the sample. Finally we consider $T(t) = \frac{1}{t_{scale}t} J_0(t_{scale}t)$, where J_0 is the Bessel function of the first kind of order 0, reported as a solution for forward propagation of time evolving acoustic pressure [1]. The upper graphs of (a, b, c) shows convoluted shapes $A_{AE}(t)$ that resembles the typical acoustic emission time profiles. However, by changing some of the parameters of the transfer functions, the convolution becomes non-oscillatory. Therefore, we choose parameters that produce the convolutions closer to the typical acoustic emission pulses measured experimentally (Fig. S2). We obtain similar scaling results for $T(t) = \cos(wt)\, e^{-qt^2}$ and $T(t) = \cos(wt)\, e^{-qt}$ and for both the convoluted waveforms are closer to the typical AE pulses. For the $T(t) = \frac{1}{t_{scale}t} J_0(t_{scale}t)$ the convoluted waveforms are oscillatory (Fig S1 c upper panel) but the avalanche profile *V(t)* is modified in the first half period and then barely changes its shape. The complete convolution analysis is shown in section 3.

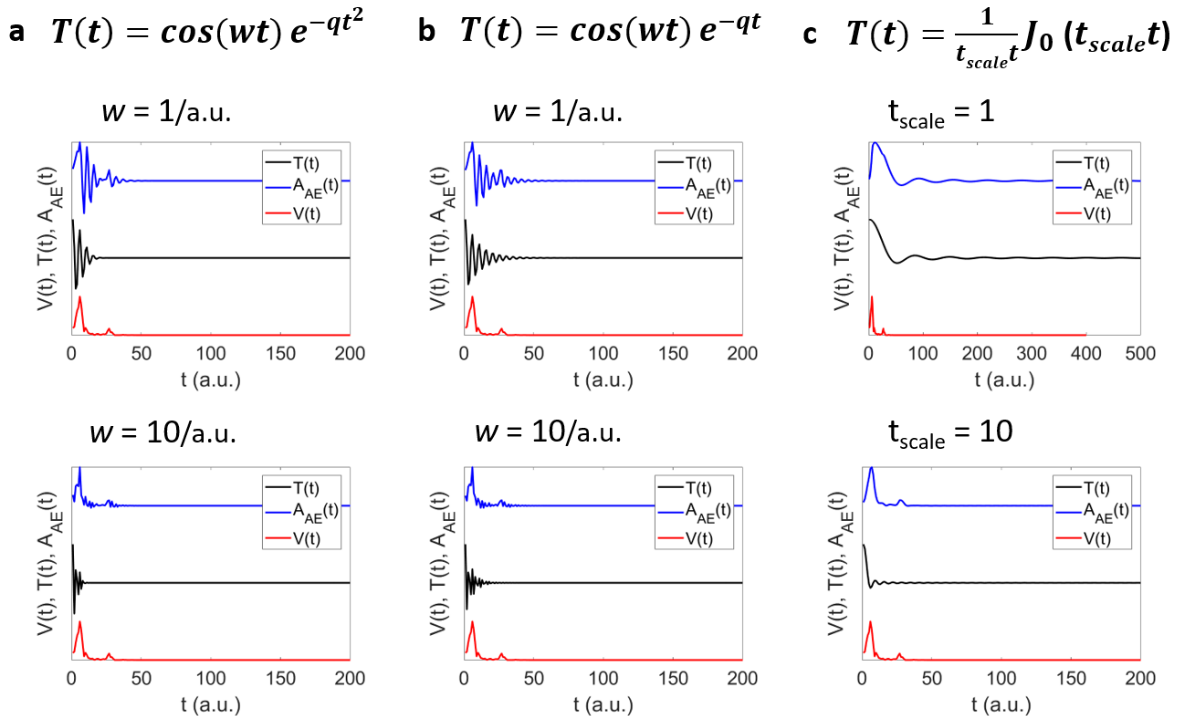

Figure S1. Example of a random selected avalanche profile V(t) from the Collapse model convoluted with different transfer functions. (a) $T(t) = \cos(wt)\, e^{-qt^2}$ at two different frequencies w= 1/a.u. and w= 10/a.u. (b) $T(t) = \cos(wt)\, e^{-qt}$ at two different frequencies w= 1/a.u. and w= 10/a.u. (c) $T(t) = \frac{1}{t_{scale}t} J_0(t_{scale}t)$ for two different time scales t_{scale}=1 and t_{scale}=10.

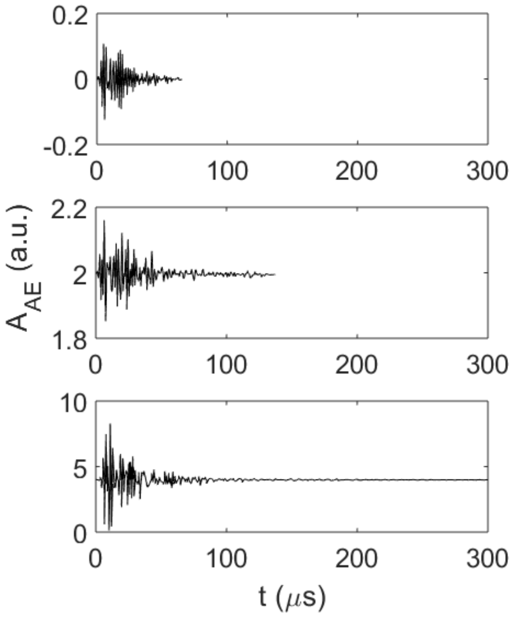

Figure S2. Three experimental AE pulses events with different durations measured on $BaTiO_3$ (111) during ferroelectric switching. Details of the measurement can be found in [2].

2. Duration with different thresholds

In Fig. S3 we show impact of the threshold on the duration D defined as $D = t(|\Psi(t \rightarrow t_n)| < \Psi th)$; where Ψth is a threshold in $\Psi(t)$ and t_n is a waiting time.

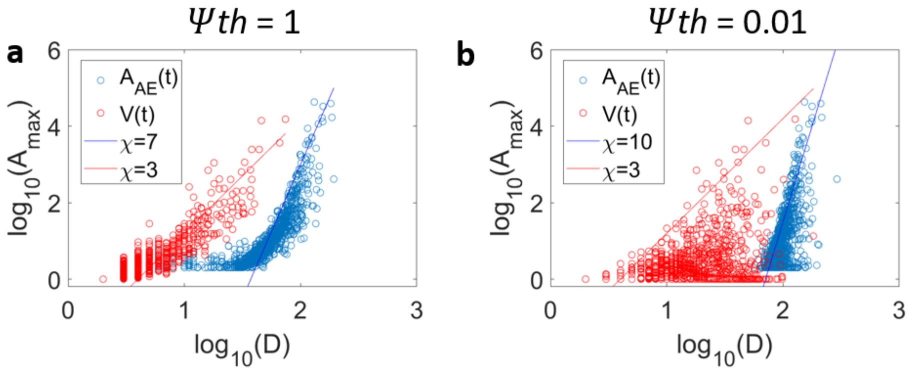

Figure S3. Duration D from the avalanche profiles *V(t)* from the collapse model and after convolution $A_{AE}(t)$ with $T(t) = \cos(wt)\, e^{-qt^2}$ computed from two different thresholds $\Psi th = 1$ and $\Psi th = 0.01$.

3. Convolution with a gaussian decay

We analysed the convolution for $T(t) = \cos(wt)\, e^{-qt^2}$ for the average avalanche source function $V(t) = ate^{-bt^2}$ by changing the b parameter (Fig. S4), for the collapse model (Fig. S5) and for the slip model (Fig. S6). In all three cases the scaling relations are very similar to the convolution with the Gaussian decay shown on the main manuscript.

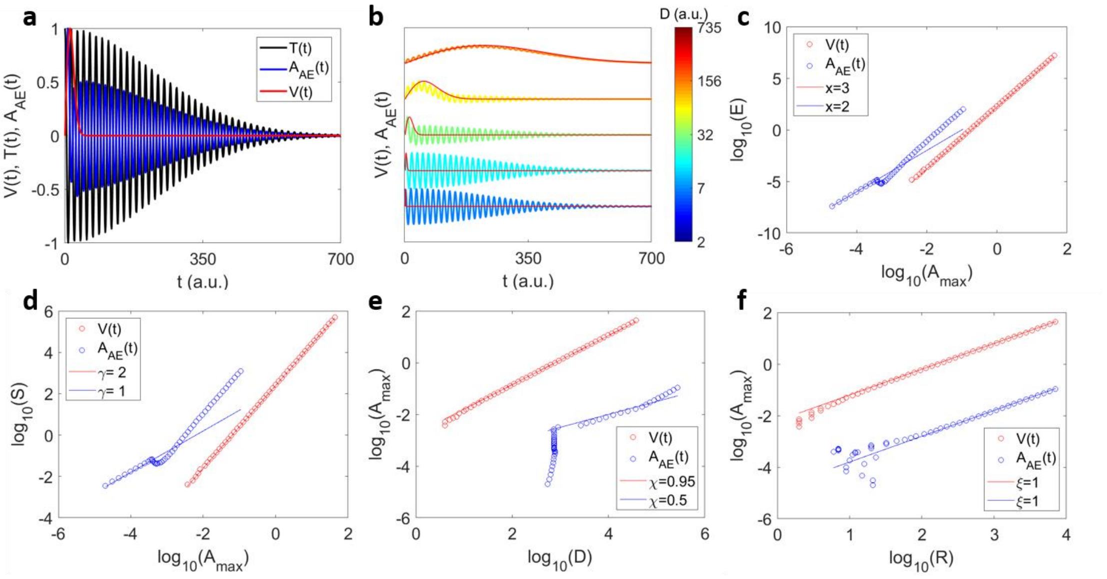

Figure S4. *V(t)* is the averaged source function $V(t) = ate^{-bt^2}$ with variable b parameter. (a) Example of convolution leading to $A_{AE}(t)$ with an avalanche profile *V(t)* (in red) and a transfer function $T(t) = \cos(wt)\, e^{-qt^2}$ (in black). (b) Examples of 6 convoluted avalanche profiles for different source functions *V(t)* with different durations, and the same transfer function. Both convoluted and original profiles are normalized with respect to their maxima. (c-f) scaling relations $E \sim A_{max}^{x}$ (c), $S \sim A_{max}^{\gamma}$ (d), $A_{max} \sim D^{\chi}$ (e) and $A_{max} \sim R^{\xi}$ (f) for the original *V(t)* profiles (red points) and after the convolution $A_{AE}(t)$ (blue points).

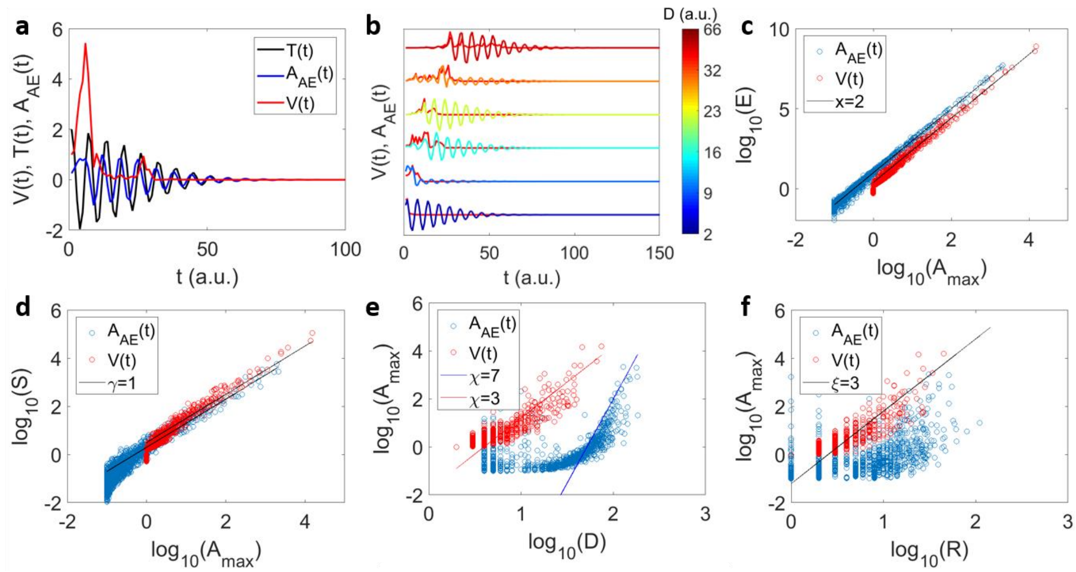

Figure S5. *V(t)* of the collapse model: (a) Example of convolution leading to $A_{AE}(t)$ with an avalanche profile *V(t)* (red) and a transfer function $T(t) = \cos(wt)\, e^{-qt^2}$ (black). (b) Examples of 6 convoluted avalanche profiles for different source functions *V(t)* with different durations, and the same transfer function. Both convoluted and original profiles are normalized with respect to their maxima. (c-f) scaling relations $E \sim A_{max}^{x}$ (c), $S \sim A_{max}^{\gamma}$ (d), $A_{max} \sim D^{\chi}$ (e) and $A_{max} \sim R^{\xi}$ (f) for the original *V(t)* profiles (red points) and after the convolution $A_{AE}(t)$ (blue points).

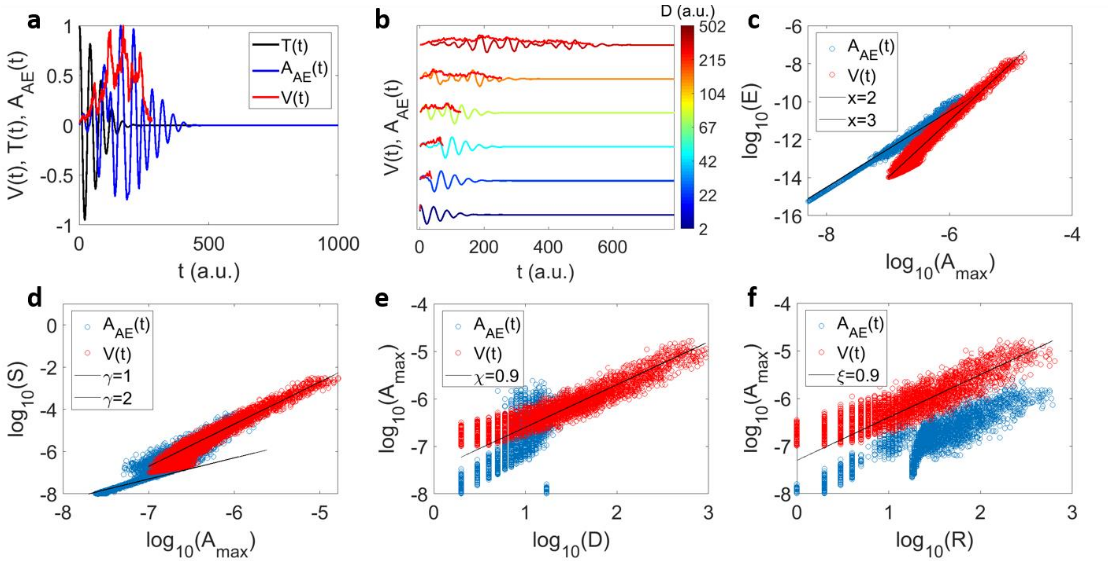

Figure S6. *V(t)* of the spin slip model: (a) Example of convolution leading to $A_{AE}(t)$ with an avalanche profile *V(t)* (red) and a transfer function $T(t) = \cos(wt)\, e^{-qt^2}$ (black). (b) Examples of 6 convoluted avalanche profiles for different source functions *V(t)* with different durations, and the same transfer function. Both convoluted and original profiles are normalized with respect to their maxima. (c-f) scaling relations $E \sim A_{max}^{x}$ (c), $S \sim A_{max}^{\gamma}$ (d), $A_{max} \sim D^{\chi}$ (e)

and $A_{max} \sim R^{\xi}$ (f) for the original *V(t)* profiles (red points) and after the convolution $A_{AE}(t)$ (blue points).

4. Convolution with Bessel functions

Here we analyse the convolution for $T(t) = \frac{1}{t}J_0(t)$ for the average avalanche source function $V(t) = ate^{-bt^2}$ by changing the b parameter (Fig. S4), for the collapse model (Fig. S5) and for the slip model (Fig. S6). In all three cases the scaling with $\chi = 1.5$ for the duration stems from the scaling of the transfer function.

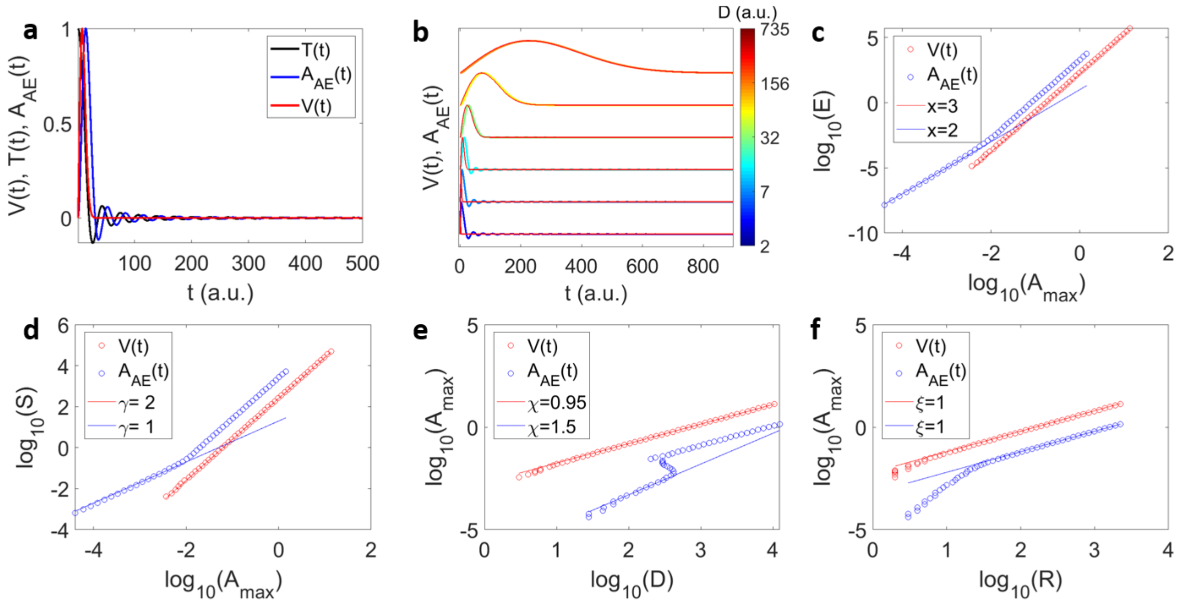

Figure S7. *V(t)* is the averaged source function $V(t) = ate^{-bt^2}$ changing the b parameter: (a) Example of convolution leading to $A_{AE}(t)$ with an avalanche profile *V(t)* (in red) and a transfer function $T(t) = \frac{1}{t}J_0(t)$ (in black). (b) Examples of 6 convoluted avalanche profiles for different source functions *V(t)* with different durations, and the same transfer function. Both convoluted and original profiles are normalized with respect to their maxima. (c-f) scaling relations $E \sim A_{max}^{x}$ (c), $S \sim A_{max}^{\gamma}$ (d), $A_{max} \sim D^{\chi}$ (e) and $A_{max} \sim R^{\xi}$ (f) for the original *V(t)* profiles (red points) and after the convolution $A_{AE}(t)$ (blue points).

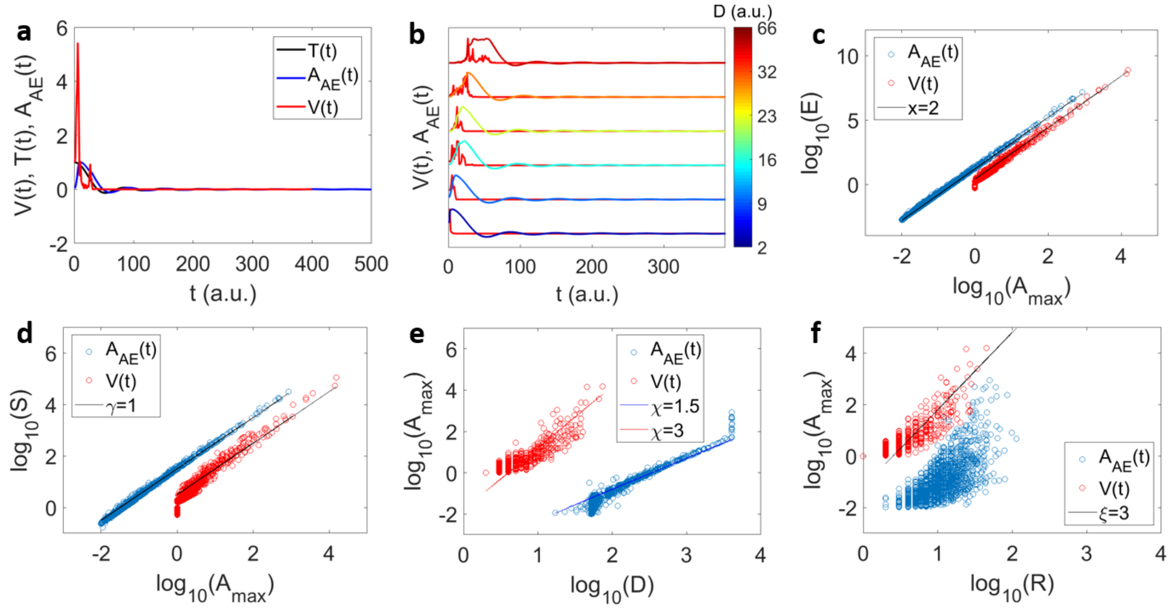

Figure S8. *V(t)* of the collapse model: (a) Example of convolution leading to *A_{AE}(t)* with an avalanche profile *V(t)* (red) and a transfer function $T(t) = \frac{1}{t} J_0(t)$ (black). (b) Examples of 6 convoluted avalanche profiles for different source functions *V(t)* with different durations, and the same transfer function. Both convoluted and original profiles are normalized with respect to their maxima. (c-f) scaling relations $E \sim A_{max}^{x}$ (c), $S \sim A_{max}^{\gamma}$ (d), $A_{max} \sim D^{\chi}$ (e) and $A_{max} \sim R^{\xi}$ (f) for the original *V(t)* profiles (red points) and after the convolution $A_{AE}(t)$ (blue points).

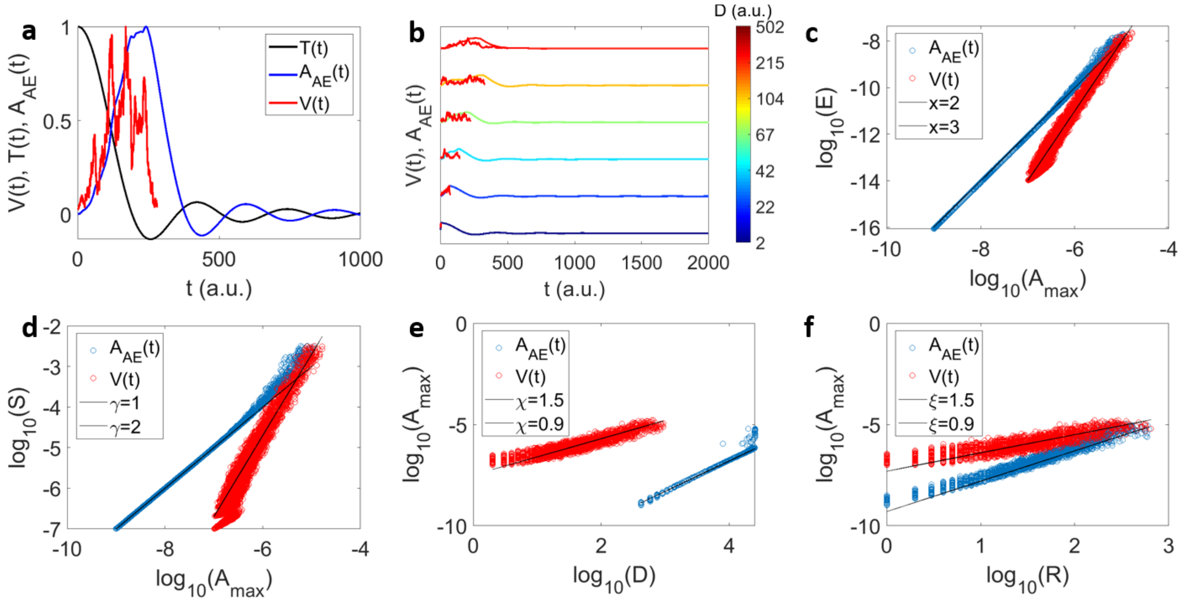

Figure S9. *V(t)* of the slip model: (a) Example of convolution leading to $A_{AE}(t)$ with an avalanche profile *V(t)* (red) and a transfer function $T(t) = \frac{1}{t} J_0(t)$ (black). (b) Examples of 6 convoluted avalanche profiles for different source functions *V(t)* with different durations, and the same transfer function. Both convoluted and original profiles are normalized with respect to their maxima. (c-

f) scaling relations $E \sim A_{max}^{x}$ (c), $S \sim A_{max}^{\gamma}$ (d), $A_{max} \sim D^{\chi}$ (e) and $A_{max} \sim R^{\xi}$ (f) for the original *V(t)* profiles (red points) and after the convolution $A_{AE}(t)$ (blue points).

5. PDFs before and after convolution

Figure S10 shows the PDFs of the avalanche magnitudes Amax, E and S, before and after convolution ($A_{max\ AE}$, E_{AE}, S_{AE}) with $T(t) = \cos(wt)\, e^{-qt^2}$.

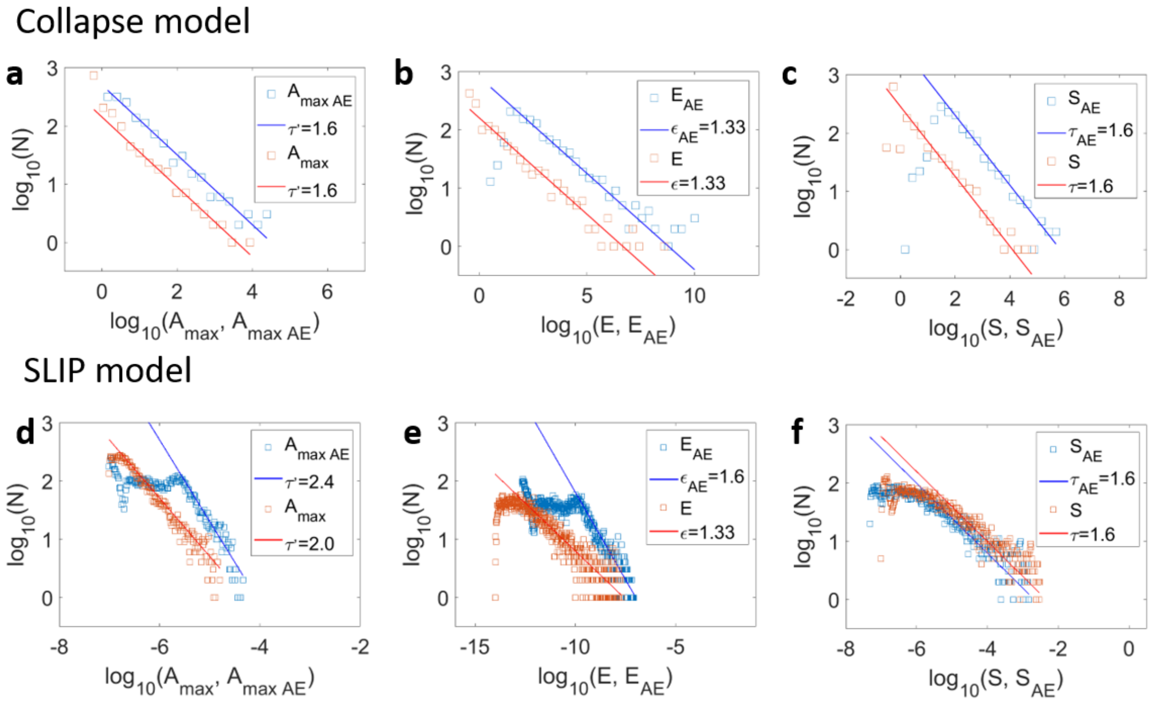

Figure S10. PDFs of the avalanches magnitudes before (A_{max}, E, S red squares) and after convolution ($A_{max\ AE}$, E_{AE}, S_{AE} blue squares) of the collapse model (a, b and c) and of the slip model (d, e and f). The convolution magnitudes have been shifted for visual reasons.

6. Average source function of the collapse model

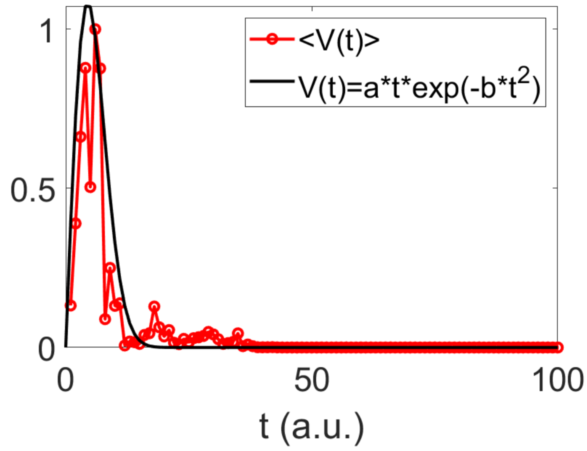

Figure S11. Average of 20 avalanches profiles obtained from the collapse model with same size (<V(t)>) and the fitting with the toy model (avalanche source function $\mathrm{V(t)} = ate^{-bt^2}$).

7. Duration and Rise Time

We now explore the PDFs of the duration *D* and the rise time *R* after convolution from the collapse model and the slip model. Fig S8. shows that the PDFs of the duration after convolution (blue points) do not preserve the scaling of the original *V(t)* (red points). The rise time scaling preserves the same power law after (green points) and before (magenta points) the convolution, however. These results show that the use of the rise time R_{AE} is an equally good, or even better, parameter to characterise the avalanches compared with the duration *D*. The rise time exponent (i.e. the power law decay exponent of the PDF of *R*) is ρ=2.2 for the collapse model (fig. S12 a) and ρ=1.8 for the slip model (fig. S8 b), both are conserved after convolution while the duration exponent (of the PDF of *D*) α varies greatly. The PDFs of A_{max}, *E*, and *S* for both models are shown in the supplementary material (section 4).

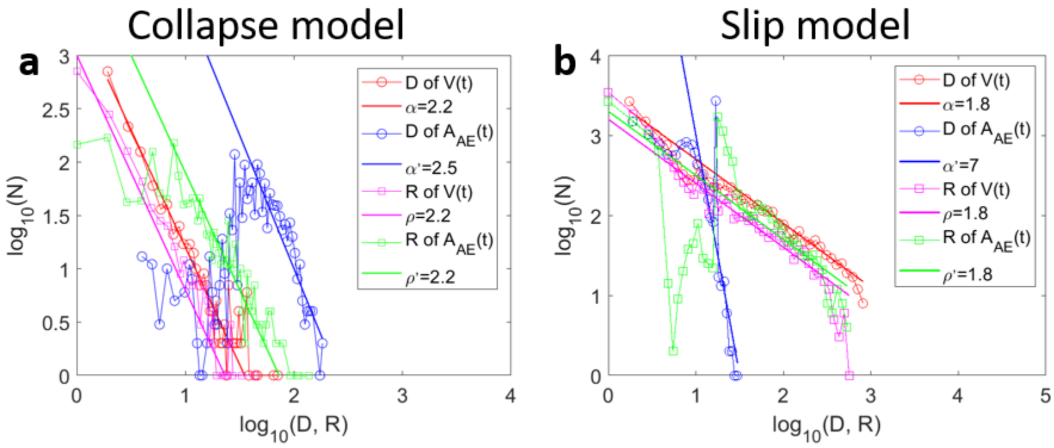

Figure S12. PDFs of the duration $PDF(D)\sim D^{\alpha}$ and the rise time $PDF(R)\sim R^{\rho}$ for the original *V(t)* profiles (red and magenta) and after the convolution $A_{AE}(t)$ (α', ρ' in green and blue) for the transfer function $\mathrm{T} = \cos(wt)\, e^{-qt^2}$. This analysis is shown for the collapse model (a) and the slip model (b).

References

[1] V. Grulier, S. Paillasseur, J-H. Thomas, J-C. Pascal, and J-C. Le Roux. J. Acoust. Soc. Am. 126 (5), November 2009

[2] E.K.H. Salje, D. Xue, X. Ding, K.A. Dahmen, J.F. Scott. Physical Review Material **3**, 014415 (2019).
